# Significance of Liver Size in Hepatic Surgery

**DOI:** 10.1155/1997/34842

**Published:** 1997

**Authors:** K. Yanaga, H. Honda, Y. Ikeda, A. T. Nishizaki, K. Yamamoto, K. Sugimachi

**Affiliations:** Department of Surgery II Kyushu University Faculty of Medicine Fukuoka Japan

## Abstract

The purpose of this study was to evaluate the significance of liver volumetry as a parameter for hepatic functional reserve in cirrhotic patients with hepatocellular carcinoma. Liver volume was calculated from preoperative computed tomograms of 44 cirrhotic patients who underwent elective hepatic resections for hepatocellular carcinoma.

The liver volume per body weight of non-alcoholic cirrhotics was significantly smaller than that of alcoholic cirrhotics (20.9 vs. 26.7 cc/kg; p=0.03). The values for alcoholic cirrhotics was comparable with normal values. The liver volume per body weight of the cirrhotic patients demonstrated correlation with the preoperative serum albumin (p<0.01) and indocyanine green clearance (p=0.02). We conclude that the determination of hepatic atrophy by volume try can serve as a parameter for the assessment of hepatic reserve but not as a predictor of postoperative complications in elective liver surgery for cirrhotic patients.


*HPB Surgery*, 1997, Vol. 10, pp. 195–200
Reprints available directly from the publisher
Photocopying permitted by license only

© 1997 OPA (Overseas Publishers Association)
Amsterdam B.V. Published in The Netherlands
by Harwood Academic Publishers
Printed in India

### Significance of Liver Size in Hepatic Surgery

K. YANAGA, H. HONDA, Y. IKEDA, A. T. NISHIZAKI, K. YAMAMOTO and K. SUGIMACHI

*Department of Surgery II, Kyushu University Faculty of Medicine, Fukuoka, JAPAN*

*(Received 5 January 1996)*

**The purpose of this study was to evaluate the significance of liver volumetry as a parameter for hepatic functional reserve in cirrhotic patients with hepatocellular carcinoma. Liver volume was calculated from preoperative computed tomograms of 44 cirrhotic patients who underwent elective hepatic resections for hepatocellular carcinoma.**

**The liver volume per body weight of non-alcoholic cirrhotics was significantly smaller than that of alcoholic cirrhotics (20.9 vs. 26.7 cc/kg; p=0.03). The values for alcoholic cirrhotics was comparable with normal values. The liver volume per body weight of the cirrhotic patients demonstrated correlation with the preoperative serum albumin (p<0.01) and indocyanine green clearance (p=0.02). We conclude that the determination of hepatic atrophy by volumetry can serve as a parameter for the assessment of hepatic reserve but not as a predictor of postoperative complications in elective liver surgery for cirrhotic patients.**

*Keywords:* Liver, hepatic resection, surgery, volumetry

### INTRODUCTION

Although liver cirrhosis used to be considered a contraindication to hepatic resection, carefully selected cirrhotic patients can now undergo elective hepatic resection safely [1–3]. The improved outcome of hepatic resection among cirrhotics has been attributed to the early detection of hepatocellular carcinomas in high risk patients, better patient selection and improved techniques for hepatic resection [1–3]. Parameters for the selection include Child's classification and indocyanine green (ICG) clearance [2–4]. With the progression of cirrhosis, the liver exhibits distortion, atrophy and impaired capacity to regenerate [5]. The progressive atrophy of the cirrhotic liver is presumably due to the loss of portal perfusion by splanchnic blood rich in hepatotrophic factors due to portal hypertension [5]. The purpose of this study was to evaluate the significance of liver volumetry as a parameter for hepatic functional reserve in cirrhotic patients with hepatocellular carcinoma.

### PATIENTS AND METHODS

During a 64 months period between April 1985 and August 1990, 123 patients underwent elective hepatic resections for hepatocellular carcinoma in the Department of Surgery II, Kyushu University Hospital. Of these, 69 (56.1%) had underlying liver cirrhosis documented by histology. Among these cirrhotic patients, 44 underwent

Correspondence to: galley proofs and reprint requests to: Katsuhiko Yanaga, M.D. Department of Surgery II, Kyushu University Faculty of Medicine, Higashi-ku, Fukuoka 812, JAPAN.

preoperative computed tomography (CT) at Kyushu University Hospital, for whom the liver volume was measured according to the methods described elsewhere [6,7]. As to the clinical characteristics of the 44 cirrhotic patients studied, their ages ranged from 42 to 75 with a mean (s.d.) of 59 (7.4), and 36 of them (81.8%) were male. Types of hepatic resection performed consisted of right lobectomy in two, left lobectomy in six, segmentectomy in six and subsegmentectomy in 30 patients. None of these patients exhibited ascites, and none demonstrated severe muscle wasting.

The etiology of underlying liver diseases was classified either alcoholic (nine patients) or non-alcoholic (35 patients, either viral or cryptogenic) based on clinical information and laboratory data. The definition of alcoholic cirrhosis consisted of the calculated yearly alcohol intake of over 86 g for more than 10 years and the absence of viral hepatitis or blood transfusion.

The liver size was compared by the calculated liver volume per body weight (LV/BW), for which reported normal values range from 20 to 27 cc/kg [6, 8]. Therefore, livers with LV/BW below 20 cc/kg were judged atrophic.

Postoperative complications consisted of subphrenic abscesses (four patients, 9.1%), bile collection (two patients, 4.5%), wound infection (11 patients, 25%), postoperative hemorrhage (one patient, 2.3%), ascites (ten patients, 22.7%), pleural effusion (10 patients, 22.7%), and liver failure (one patient, 2.3%). Hospital death occurred in two cirrhotic patients (4.5%), one alcoholic and one non-alcoholic. Statistical analysis was by the two-tailed Student's t test and chi-square test with Yate's correction.

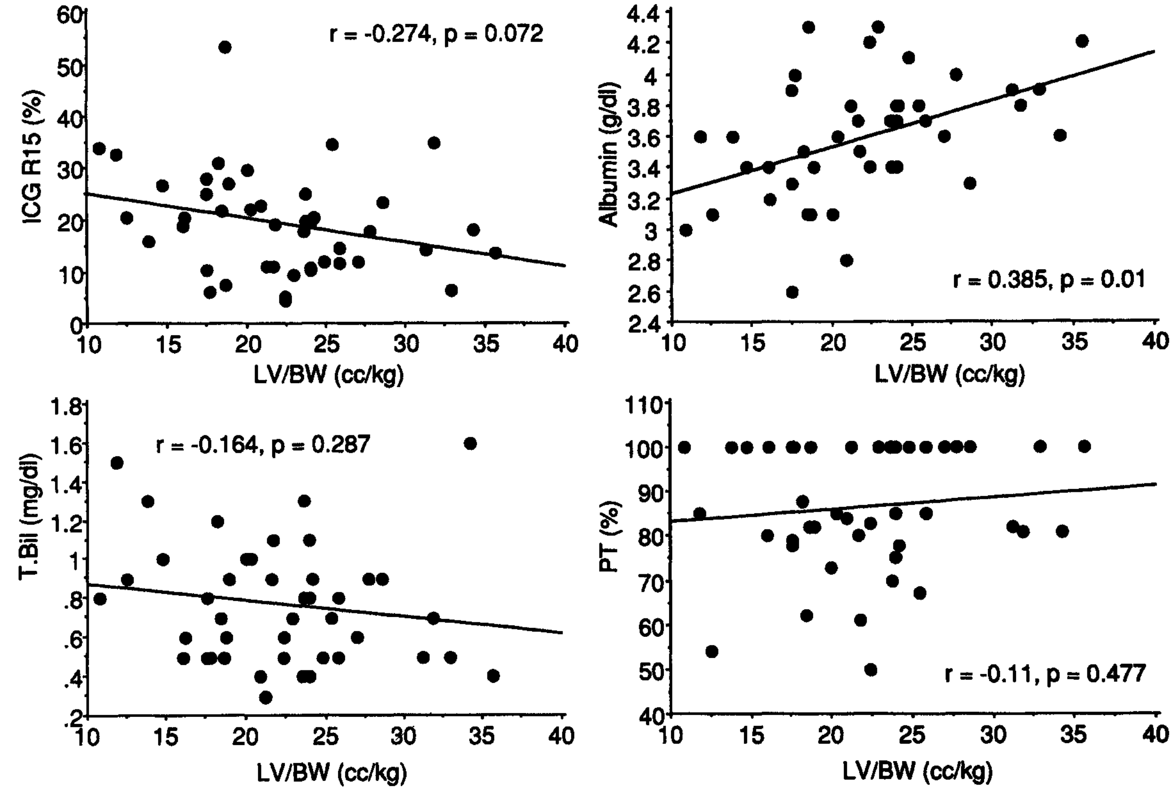

FIGURE 1 Liver size in relation to conventional preoperative data. None of the parameters demonstrated a significant correlation with the liver size. LV/BW = liver weight per body weight, ICG R15 = indocyanine green retention rate at 15 min, T. Bil = serum total bilirubin, PT = prothrombin time.

### RESULTS

Figure 1 exhibits the correlation between LV/ BW and conventional parameters of liver function. None of such preoperative variables showed a significant correlation with the LV/BW.

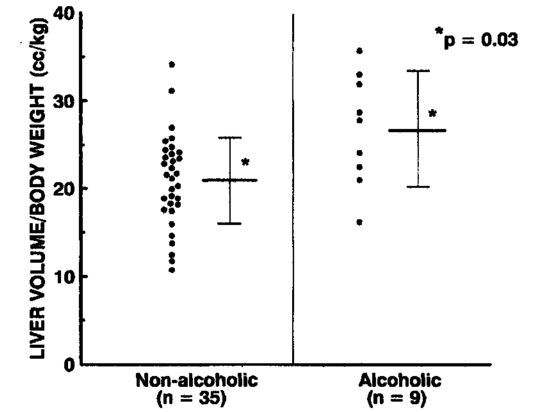

FIGURE 2 Liver size in relation to the etiology of liver diseases among cirrhotic patients who underwent hepatic resection. The liver size of alcoholic patients were significantly larger than that of non-alcoholics ($P$=0.03).

Figure 2 demonstrates LV/BW of alcoholic and non-alcoholic patients who underwent hepatic resection. Only one of the nine patients (10%) with alcoholic cirrhosis had LV/BW below 20 cc/ kg, and LV/BW was significantly larger among alcoholic cirrhotics as compared to non-alcoholics ($P$=0.03). The LV/BW of alcoholic cirrhotics were comparable to that of normal subjects [6,8].

Table I compares preoperative variables of cirrhotic patients who underwent hepatic resection between those with LV/BW below or above 20 cc/ kg. For those with LV/BW of below 20 cc/kg, the serum albumin and the ICG retention rate were significantly worse ($P$<0.01 and $P$=0.02, respectively) than ones with larger livers. Other variables were comparable between the two groups.

Table II compares the two groups in relation to intra- or postoperative variables. The types of resection and resection rate were comparable between the two groups, which is consistent with our policy of not using liver size as a preoperative factor for determining the type of resection. Patients with smaller livers tended to have higher portal pressure but smaller amount of blood loss and shorter operation time, which did not achieve statistical significance. Other variables, including the tumor diameter, postoperative complications and hospital mortality were comparable between the two groups.

TABLE I Relationship between preoperative liver volume and other preoperative variables among cirrhotic patients who underwent hepatic resection

| | *Liver volume/BW (cc/kg)* | | *P* |
|---|---|---|---|
| | < 20 (*n*=17) | ≥ 20 (*n*=27) | |
| Age (years) | 60.2 ± 8.8[a] | 58.1 ± 6.5 | 0.41 |
| Sex (M:F) | 13 : 4 | 23 : 4 | 0.74 |
| Albumin (g/dl) | 3.21 ± 0.88 | 3.72 ± 0.31 | <0.01 |
| T. Bil (mg/dl) | 0.83 ± 0.3 | 0.73 ± 0.31 | 0.31 |
| PT(%) | 86.1 ± 14.4 | 86.9 ± 14.1 | 0.85 |
| ICG(%) | 24.3 ± 11.3 | 16.6 ± 7.7 | 0.02 |
| Platelets (x $10^3/mm^3$) | 116.4 ± 52.3 | 129.1 ± 57.3 | 0.46 |
| Cr (mg/dl) | 0.99 ± 0.37 | 0.92±0.25 | 0.47 |

[a]mean ± s.d. BW=body weight. T. Bil=total bilirubin. PT prothrombin time. ICG=indocyanine green retention rate at 15 min (normal < 10%). Cr=creatinine.

TABLE II Relationship between preoperative liver volume and intra- or postoperative variables among cirrhotic patients who underwent hepatic resection

| | Liver volume/BW (cc/kg) | | *P* |
|---|---|---|---|
| | < 20 (*n*=17) | ≥ 20 (*n*=27) | |
| Portal pressure ($cmH_2O$) | 25.2 ±6.9[a] | 22.7±4.2 | 0.14 |
| Type of resection[b] | 6/1/10 | 2/5/20 | 0.47[c] |
| Resection rate (g/cc)[d] | 0.13 ± 0.14 | 0.14 ± 0.13 | 0.95 |
| Tumor diameter (cm) | 2.9 ± 1.31 | 3.37 ± 1.9 | 0.35 |
| Operation time (min) | 264 ± 74 | 312 ± 98 | 0.07 |
| EBL (cc) | 1039 ± 649 | 1630 ± 1200 | 0.07 |
| Postoperative hospital stay (days) | 27.9 ± 11.1 | 32.1 ± 24.3 | 0.50 |
| Complications | 6 (35.3%) | 13 (48.1%) | 0.60 |
| Hospital death | 1 ( 5.9%) | 1 ( 3.7%) | 1.00 |

[a]mean ± s.d. EBL = estimated blood loss.
[b]lobectomy/segmentectomy/subsegmentectomy.
[c]lobectomy and segmentectomy vs. subsegmentectomy.
[d]resected weight/calculated liver weight.

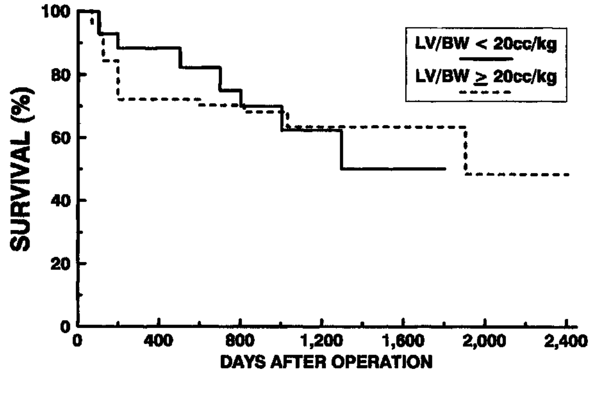

FIGURE 3 Survival after hepatic resection among cirrhotic patients in relation to the liver size. There were no differences between the two groups.

Figure 3 demonstrates the survival after hepatic resection among the cirrhotic patients in relation to the liver size. The survival was not affected by the liver size.

### DISCUSSION

It is well-known that the livers of patients with advanced cirrhosis exhibit progressive atrophy [9]. This study demonstrated a significant difference in the liver size by the etiology of liver diseases. To date, little is known about the relationship between the etiology of liver diseases and hepatic atrophy. Henderson *et al.* in 1983 [10] reported hemodynamic differences between alcoholic and nonalcoholic cirrhotics after distal splenorenal shunt, in which the mean liver size calculated by CT tended to be, but was not significantly smaller in eight non-alcoholic cirrhotics as compared to 16 alcoholic cirrhotics (1,489 vs. 2,113 cc). El-Khishen *et al.* in 1985[11] reported among cirrhotics who underwent distal splenorenal shunt that hypersplenism was more common in non-alcoholics than in alcoholics, while Yanaga *et al.* in 1989 [12] documented among advanced postnecrotic cirrhotics who underwent orthotopic liver transplantation that the incidence of hypersplenism was the same for the two groups, although it has been known among transplant surgeons that cirrhotic livers of alcoholic etiology are often large at the time of transplant.

The positive correlation between the liver size and established preoperative parameters of hepatic functional reserve, i.e., ICG and albumin [1–4], suggest that the liver volume determination from preoperative CT could also be used as a parameter for the assessment of liver function. However, hepatic atrophy *per se* in our series was not associated with increased mortality or morbidity after hepatic resection. We assume that, for the patients with small livers, an appropriate inflow control and a faster parenchymal division than for normal- or large-sized livers account for smaller blood loss and operation time which helped reduce the mortality and morbidity after hepatic resection among cirrhotics. We continue to believe that the resection rate is more important than the absolute resection volume in elective hepatic resection for cirrhotic patients.

As to the significance of volumetry in liver surgery, Okamoto *et al.* in 1984 [13] reported that preoperative estimation of the resection rate of non-cancerous rather than the whole hepatic parenchymal volume might be able to predict post-hepatectomy liver failure. With better understanding of high risk patients and improved diagnostic modalities, hepatocellular carcinomas are now detected at an early stage [1,2]. Since the tumor size at the time of detection is much smaller as in our series, it seems simpler to evaluate the entire liver volume for the assessment of hepatic atrophy under such a circumstance.

### *References*

[1] Matsumata, T., Kanematsu, T., Shirabe, K., Sonoda, T., Furuta, T. and Sugimachi, K. (1990). Decreased morbidity and mortality rates in surgical patients with hepatocellular carcinoma. *British Journal of Surgery*, 77, 677–680.
[2] Belghiti, J. (1991). Resection of hepatocellular carcinoma complicating cirrhosis. *British Journal of Surgery*, **78**, 257–258.
[3] MacIntosh, E.L. and Minuk, G.Y. (1992). Hepatic resection in patients with cirrhosis and hepatocellular carcinoma. *Surgery, Gynecology and Obstetrics*, **174**, 245–254.
[4] Hemming, A.W., Scudamore, C.H., Shackleton, C.R., Pudek, M, and Erb, S.R. (1992). Indocyanine green clearance as a predictor of successful hepatic resection in cirrhotic patients. *American Journal of Surgery*, **163**, 515–518.
[5] Sherlock, S. (1989). The portal venous system and portal hypertension. In Diseases of the liver and biliary system, edited by Sherlock S, pp. 151–207, Oxford: Blackwell Scientific Publications.
[6] Henderson, J.M., Heymsfield, S.B., Horowitz, J, and Kutner, M.H. (1981). Measurement of liver and spleen volume by computed tomography: assessment of reproducibility and changes found following a selective splenorenal shunt. *Radiology*, **141**, 525–527.
[7] Honda, H., Onitsuka, H., Masuda, K., Nishitani, H., Nakata, K, and Watanabe, K. (1990). Chronic liver disease: value of volumetry of liver and spleen with computed tomography. *Radiation Medicine*, **8**, 222–226.
[8] Stieber, A.C., Makowka, L, and Starzl, T.E. (1992). Orthotopic liver transplantation. In Atlas of Organ Transplantation, edited by Starzl, T.E., Shapiro, R., Simmons, R.L., pp. 71–51, New York: Gower.
[9] Van Thiel, D.H., Makowka, L, and Starzl, T.E. (1988). Liver transplantation: where it's been and where it's going. *Gastroenterology Clinics of North America*, **17**, 1–18.
[10] Henderson, J.M., Millikan, W.J. Jr., Wright-Bacon, L., Kutner, M.H, and Warren, W.D. (1983). Hemodynamic differences between alcoholic and nonalcoholic cirrhotics following distal splenorenal shunt: effect on survival? *Annals of Surgery*, **198**, 325–334.
[11] El-Khishen, M.A., Henderson, J.M., Millikan, W.J. Jr., Kutner, M.H, and Warren, W.D. (1985). Splenectomy is contraindicated for thrombocytopenia secondary to portal hypertension. *Surgery, Gynecology and Obstetrics*, **160**, 233–238.
[12] Yanaga, K., Tzakis, A.G., Shimada, M., Campbell, W.E., Marsh, J.W., Makowka, L., Todo, S., Iwatsuki, S, and Starzl, T.E. (1989). Reversal of hypersplenism following orthotopic liver transplantation. *Annals of Surgery*, **210**, 180–183.
[13] Okamoto, E., Yamanaka, N., Tanaka, N, and Kuwata, K. (1984). Prediction of the safe limits of hepatectomy by combined volumetric and functional measurements in patients with impaired hepatic function. *Surgery*, **95**, 586–592.

### COMMENTARY

Because of the important functional reserve of the liver and its ability to regenerate, resection of up to 6 segments (as defined by Couinaud) can be tolerated by most patients without underlying liver disease. In contrast, patients with an underlying liver disease and in particular liver cirrhosis, are at increased risk of in-hospital mortality and morbidity as a consequence of overt or latent postoperative liver failure even if these patients have no ascites and have normal bilirubin, albumin and prothrombin time at the time of surgery (Child grade A). This is, at least in part, related to the poor "functional reserve" of cirrhotic livers and to an impaired ability of these livers to undergo regeneration.

Accurate assessment of this functional reserve is currently lacking in these patients with well compensated cirrhosis. Sophisticated tests such as the bromosulphalein or the indocyanin green clearance, the glucose tolerance test, the redux tolerance index or a combination of these have been advocated and are used in Eastern countries. However these tests are not always easily available, may be influenced by the functional hepatic blood flow or minor degrees of biliary obstruction, so that their influence on the postoperative course has not been confirmed.

On the other hand, the mechanisms responsible for liver regeneration (and the reason why cirrhotic livers have impaired regeneration capacity) remain unclear. Another approach has therefore been to try to increase, prior to resection, the volume of liver parenchyma anticipated to be spared by the resection through the selective portal embolization of the liver segments anticipated to be involved in the resection. There is indeed growing evidence that even a cirrhotic liver may undergo hypertrophy through this process.

For these reasons, the study of Yanaga *et al.* assessing the correlation between liver volume on the one hand and liver function as well as outcome after liver resection on the other hand is timely. The authors show that there is a correlation between the preoperative size of the liver (expressed as the ratio of liver volume to body weight) and both preoperative ICG clearance and serum albumin levels (i.e. that patients with small livers tend to have low serum albumin levels and high ICG retention rates), and most importantly that patients with small livers (defined as a ratio of liver volume to body weight less than 20 cc/kg) have a postoperative outcome comparable to that of patients with larger livers (i.e. that some degree of liver atrophy is not necessarily associated with an unfavourable postoperative course).

Based on these results, what may be the practical applications of liver volumetry in the future? We believe that the method of volume assessment may first need some adjustments. In the present study, the liver volume was expressed in relation to the body weight. Indeed, the liver volume per se is of little value. However, recent experience with the split liver and living related donor transplantations suggest that the "optimal" liver volume is not so much related to the weight of its recipient than to the body surface. This is probably particular true in cirrhotic patients in whom malnutrition or the presence of ascites may otherwise bias the results. It may, in addition, prove useful to combine the volumetric and the functional assessment of the liver. Indeed, experience with liver transplantation shows that the risk of primary non-function or poor function is not so much related to the small size of the graft than to an associated injury such as steatosis or related to prolonged ischaemia. Finally, what is probably relevant is not so much the volume of the whole liver at the time of surgery than the volume of liver remaining after the hepatectomy. Further studies are obviously welcome in this area of research.

J. Belghiti and O. Farges
Department of Digestive Surgery
Hospital Beaujon
F-92110 CLICHY
University Paris VII

